# Current Evidence, Controversies, and the Future of Biotics in the Prevention of Necrotizing Enterocolitis: A Narrative Review

**DOI:** 10.3390/children13080978

**Published:** 2026-07-23

**Authors:** Harshkumar R. Patel, Mohan Pammi

**Affiliations:** Department of Pediatrics, Division of Neonatology, Baylor College of Medicine and Texas Children’s Hospital, 6621 Fannin Street, Houston, TX 77030, USA; harshkumar.patel@bcm.edu

**Keywords:** preterm, neonate, necrotizing enterocolitis, biotics, probiotics, prebiotics, synbiotics, postbiotics

## Abstract

**Highlights:**

**What are the main findings?**

- Multi-strain probiotic formulations, particularly those combining *Lactobacillus* and *Bifidobacterium* species, significantly reduce the incidence of necrotizing enterocolitis (NEC) and all-cause mortality in very-low-birth-weight (VLBW) infants.
- While prebiotics alone show limited impact on NEC prevention, synbiotics (combining probiotics and prebiotics) may offer superior protection against severe NEC and improve feeding tolerance compared to probiotics alone.

**What are the implications of the main findings?**

- Widespread clinical adoption of biotics is currently limited by regulatory challenges, variable product quality, and a significant evidence gap regarding the safety and efficacy of these interventions in extremely low-birth-weight (ELBW) infants.
- The future of NEC prevention lies in precision microbiome approaches, where personalized therapies are informed by multi-omics profiling and administered via advanced delivery systems like nanoencapsulation or biofilm-based technologies.

**Abstract:**

Necrotizing enterocolitis (NEC) remains one of the most devastating gastrointestinal disorders affecting preterm infants, with substantial mortality and long-term morbidity despite advances in neonatal care. Increasing evidence implicates intestinal dysbiosis, impaired intestinal barrier function, and dysregulated immune responses as central drivers of NEC pathogenesis, making microbiome-targeted interventions a promising preventive strategy. Collectively termed “biotics,” these interventions include probiotics, prebiotics, synbiotics, and postbiotics, each aimed at modulating the developing gut ecosystem. This narrative review summarizes current evidence on the efficacy, safety, and clinical applicability of biotics for NEC prevention in very preterm and very-low-birth-weight infants. Randomized controlled trials and meta-analyses involving more than 10,000 infants demonstrate that specific multi-strain probiotic formulations, particularly those combining *Lactobacillus* and *Bifidobacterium* species, reduce NEC incidence and all-cause mortality, although benefits are less consistent in extremely low-birth-weight infants. Prebiotics alone showed a limited impact on NEC prevention, while emerging evidence suggests synbiotics may offer additive or superior protection compared with probiotics alone. Postbiotics represent a novel and potentially safer alternative, especially for the most vulnerable infants, though clinical data remain limited. Despite favorable effectiveness in meta-analyses, probiotics adoption remains variable due to strain heterogeneity, variable product quality, regulatory challenges, and rare but serious safety concerns. Precision microbiome approaches, pharmaceutical-grade formulations, personalized therapies informed by multi-omics profiling, and next-generation delivery systems may drive the future in this field.

## 1. Introduction

### 1.1. Epidemiology and Clinical Significance of Necrotizing Enterocolitis in Preterm Infants

Necrotizing enterocolitis (NEC) is fundamentally a disease of prematurity, and infants born with birth weight less than 1000 g (extremely low birth weight, ELBW) are at particularly high risk. NEC remains the most devastating gastrointestinal condition affecting preterm neonates, with high mortality rates ranging from 20 to 30% in typical cases and increasing to 30–50% in more severe cases [1]. The incidence of NEC inversely correlates with gestational age and birth weight, affecting approximately 7–10% of very-low-birth-weight (VLBW, <1500 g) infants and up to 12% of ELBW (<1000 g) infants [1,2]. Despite therapy, NEC imposes substantial burdens on survivors, including neurodevelopmental impairment, short bowel syndrome, and growth failure [3,4].

### 1.2. Pathogenesis Overview

**Figure 1:**
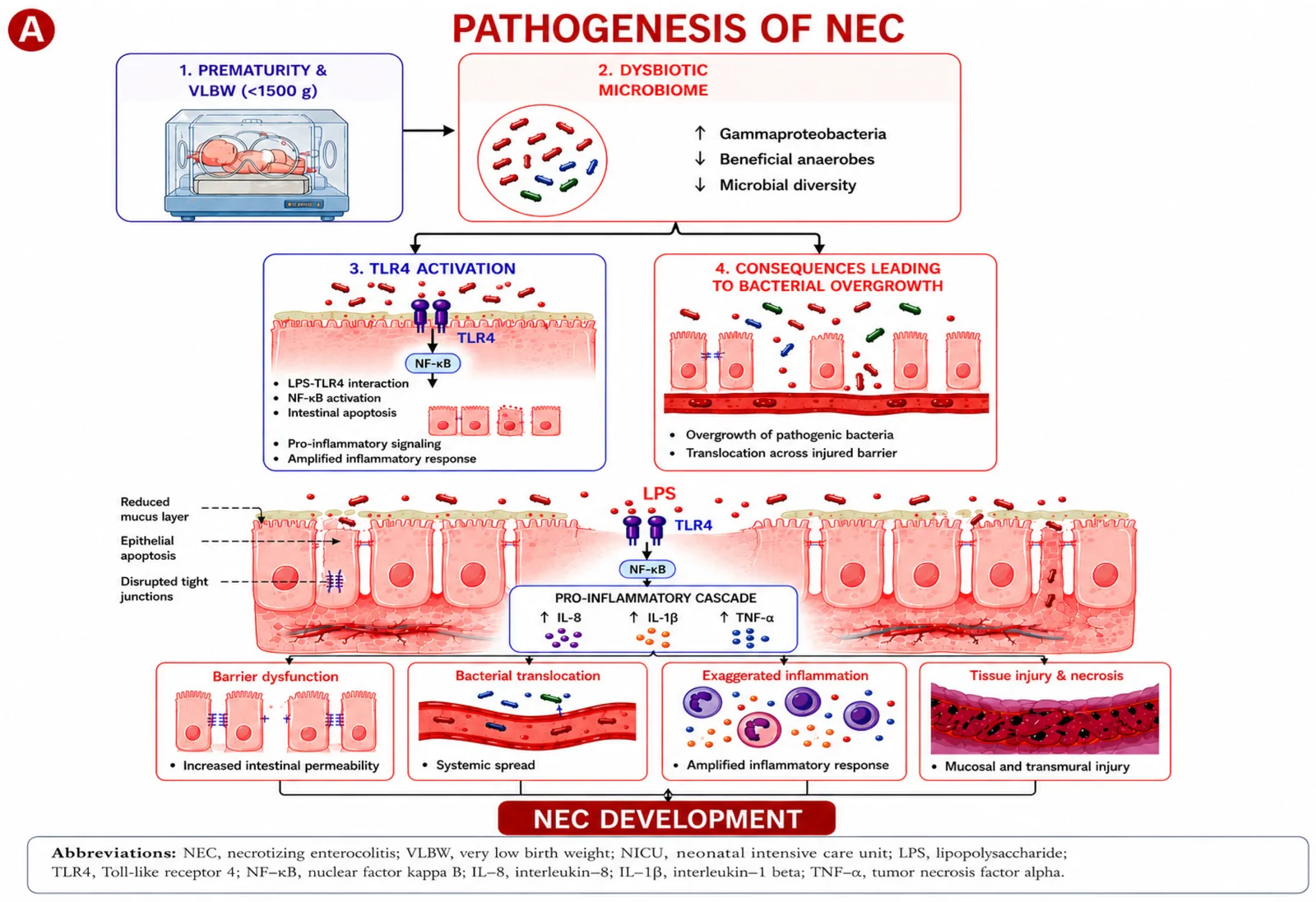
Pathogenesis of necrotizing enterocolitis (NEC). Intestinal dysbiosis, characterized by increased Gammaproteobacteria, reduced beneficial anaerobic commensals, and decreased microbial diversity, promotes bacterial lipopolysaccharide (LPS)-mediated activation of Toll-like receptor 4 (TLR4) in the immature intestine. Enhanced TLR4 signaling activates nuclear factor kappa B (NF-κB), leading to increased production of interleukin (IL)-8, IL-1β, and tumor necrosis factor-alpha (TNF-α). Persistent inflammatory signaling causes epithelial injury, promotes bacterial translocation, necroptosis, epithelial sloughing, and intestinal necrosis, culminating in NEC. The figure was conceptually designed by the authors with artificial intelligence-assisted illustration support from ChatGPT Image 1 by OpenAI.

NEC pathogenesis is multifactorial, involving interactions between intestinal immaturity, abnormal microbial colonization, and dysregulated inflammation [1,5,6,7]. Intrauterine growth retardataion, and maternal chorioamnionitis are associated with increased risk of developing NEC [8,9,10]. Significant research over the last decade has highlighted the central role of intestinal dysbiosis characterized by reduced microbial diversity, overgrowth of pathogenic Gammaproteobacteria, and depletion of beneficial anaerobes in NEC development [11,12]. This dysbiosis state, combined with the immature intestinal barrier and exaggerated innate immune responses in preterm infants, creates a perfect storm for intestinal injury [5,7,13,14]. Although the precise sequence of initiating events remains incompletely understood, experimental evidence from animal models and in vitro studies suggests that enterocyte apoptosis, driven by TLR4-mediated signaling in the immature epithelium, represents an early pathological event that precedes bacterial translocation and the amplification of intestinal inflammation [15,16]. The proposed pathogenic cascade is summarized in Figure 1. In extremely premature infants, limited physiological reserves across organ systems renders intestinal injury a potent trigger of multisystem deterioration, underscoring the importance of preventive strategies.

### 1.3. Rationale for Microbiome-Targeted Therapies

Understanding the pathophysiology of NEC provides critical context for evaluating microbiome-modulating therapies, collectively known as “biotics”. This category includes probiotics (live beneficial microbes), prebiotics (beneficial substrates), synbiotics (Probiotics and Prebiotics), and postbiotics (non-viable components from probiotic bacteria) [17,18]. Although more than 10,000 preterm infants have participated in randomized controlled trials evaluating these therapies [14], their routine use in neonatal intensive care units (NICUs) remains controversial, with significant global practice variation [19].

This narrative review synthesizes evidence on probiotics, prebiotics, synbiotics, and postbiotics for NEC prevention. It examines their therapeutic rationale, addresses adoption controversies, and identifies future research priorities to advance this field. This manuscript was developed by starting with the most recent Cochrane systematic reviews published on biotics and NEC prevention, and subsequently pearling references and conducting related article searches in PubMed. Priority was given to randomized controlled trials, network meta-analyses, systematic reviews, and international clinical guidelines. Studies were selected based on relevance to the review objectives, methodological quality, and recency, with no formal date restriction applied.

### 1.4. Microbiome and Pathogenesis of NEC

Preterm gut colonization has been shown to progress from Bacilli to Gammaproteobacteria to Clostridia [20]. A study of 166 VLBW infants found significantly higher Gammaproteobacteria and lower Negativicutes and the combined Clostridia–Negativicutes class after one month of age, with the strongest association in infants <27 weeks’ gestation [21]. NEC has been shown to be preceded by Proteobacterial bloom and Gammaproteobacteria expansion [11], and relative depletion of strict anaerobes [21,22]. Metagenomic sequencing studies have implicated colonization with specific *Escherichia coli* subtypes as a risk factor for NEC [22]. Dysbiosis detectable within the first 72 h, marked by low Actinobacteria (*Bifidobacterium*) and decreased alpha diversity, correlates with perinatal inflammation and increased NEC risk [23]. The dysbiotic microbiome is associated with NEC metabolic alterations. Depletion of *Bifidobacterium* reduces short-chain fatty acids (SCFAs) essential for barrier integrity and immune homeostasis, fostering an inflammatory environment [12,24].

Antibiotic exposure, feeding practices, delivery mode, and the NICU environment substantially influence early microbial assembly. Antibiotics reduce microbial diversity while promoting pathobionts, with prolonged courses exerting long-lasting effects. Human milk feeding reduces NEC risk and promotes colonization with beneficial taxa supported by human milk oligosaccharides (HMOs). Cesarean delivery and NICU exposure further contribute to delayed and hospital-adapted colonization patterns [6,19,25,26,27].

## 2. Probiotics

Probiotics are “live microorganisms that, when administered in adequate amounts, confer a health benefit to the host”. In NEC prevention, they act through pathogen exclusion, barrier fortification, immune modulation, and metabolite production [28].

### 2.1. Evidence from Randomized Controlled Trials

Three early randomized trials conducted outside the United States demonstrated significant reductions in NEC incidence with probiotic supplementation. Bin-Nun et al. (Israel) [29] evaluated a mixture of *B. infantis*, *S. thermophilus*, and *B. bifidus*; Dani et al. (Italy) [30] evaluated *L. rhamnosus GG*; and Lin et al. (Taiwan) [31] evaluated *L. acidophilus* and *B. bifidum.* The large multicenter trials have yielded mixed results, particularly in the highest-risk populations. The PiPS trial, conducted in the United Kingdom, randomized 1315 very preterm infants to *B. breve* supplementation and found no difference in NEC (RR 0.93, 95% CI 0.68–1.27), sepsis (RR 0.97, 95% CI 0.73–1.29), or death (RR 0.93, 95% CI 0.67–1.30) before hospital discharge. The ProPrems trial in Australia and New Zealand evaluated a probiotic combination (*B. infantis*, *S. thermophilus*, and *B. lactis*) in 1099 VLBW infants. While the overall incidence of NEC was reduced (2.0% vs. 4.4%; RR 0.46, 95% CI 0.23–0.93), a pre-specified subgroup analysis of infants born at 28 weeks’ gestation and with birth weight 1000 g showed no difference in NEC rates [19,32,33].

### 2.2. Evidence from Systematic Reviews and Meta-Analyses

**Figure 2:**
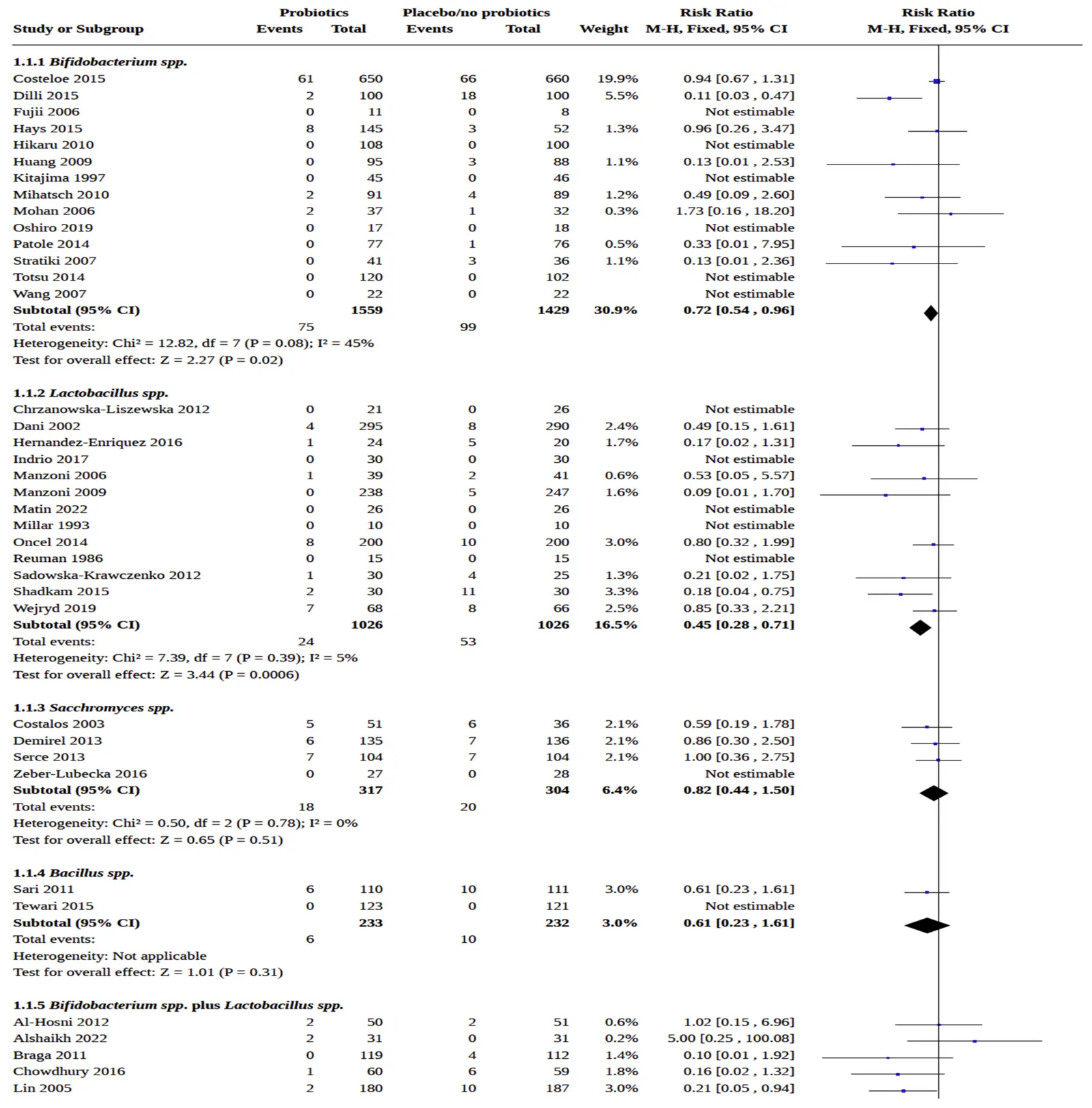

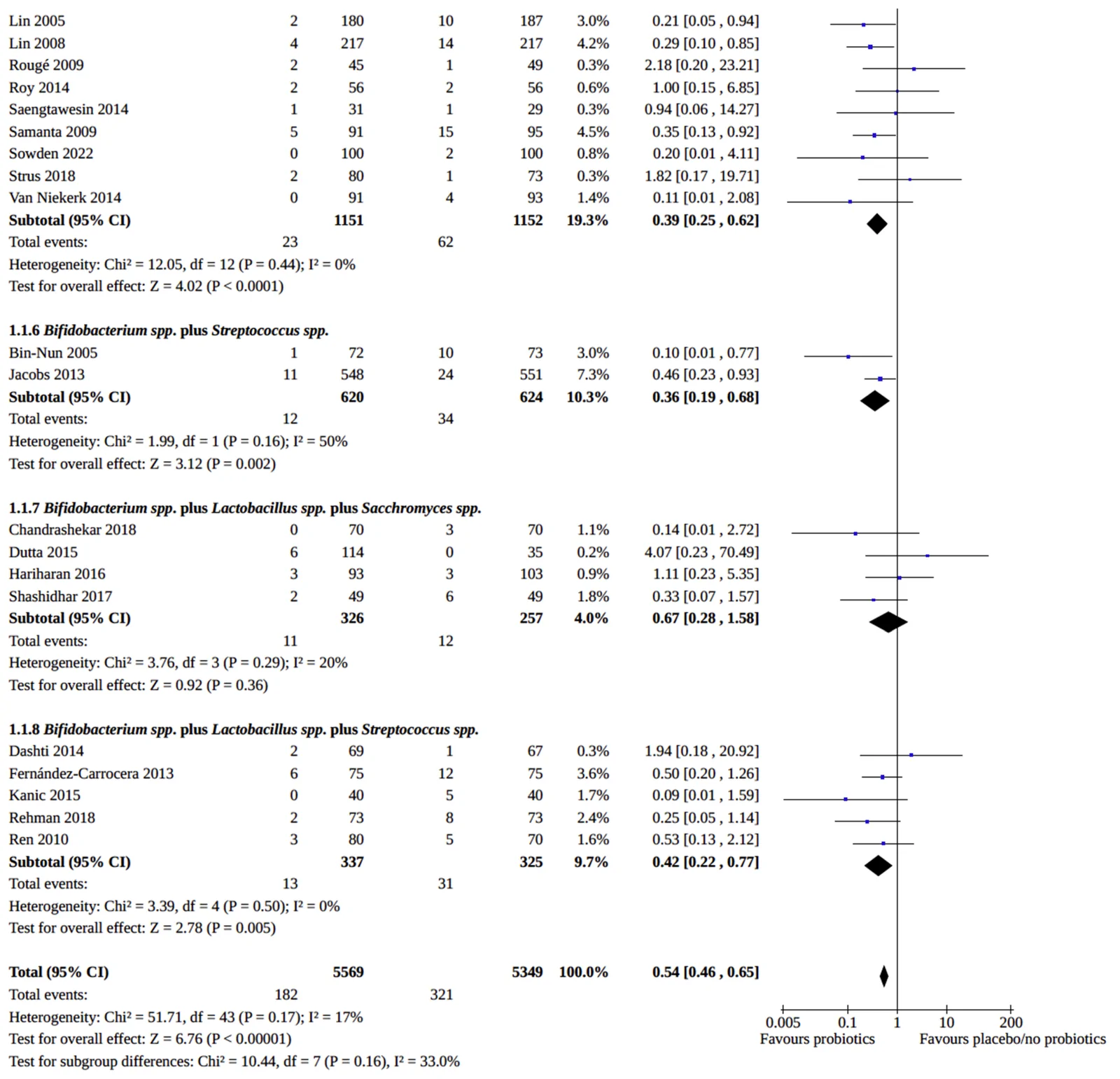
Forest plot showing the effect of probiotic supplementation on NEC risk in preterm infants. Forest plot of randomized controlled trials evaluating the efficacy of probiotic supplementation for prevention of NEC in preterm and very-low-birth-weight infants. Probiotic regimens included single-strain preparations (*Bifidobacterium* spp., *Lactobacillus* spp., and *Saccharomyces* spp.) and multi-strain combinations containing *Bifidobacterium*, *Lactobacillus*, *Streptococcus*, and *Saccharomyces* species. Risk ratios (RRs) with 95% confidence intervals (CIs) were calculated using a fixed-effects model. Overall, probiotic supplementation significantly reduced NEC risk compared with placebo or no probiotics (RR 0.54, 95% CI 0.46–0.65), with the greatest benefit observed in multi-strain formulations containing *Bifidobacterium* and *Lactobacillus* species. Adapted from a published Cochrane Collaboration systematic review and meta-analysis of probiotics for prevention of NEC in preterm infants [12].

Pooled risk ratios from over 10,000 infants strongly favor probiotics for NEC prevention. A 2023 Cochrane review found that effective formulations varied considerably across the 60 included trials (11,156 infants), with the most common preparations containing *Bifidobacterium* spp., *Lactobacillus* spp., *Saccharomyces* spp., and *Streptococcus* spp., alone or in combination. The review found that probiotics reduce NEC incidence (RR 0.54, 95% CI 0.46–0.65, low-certainty evidence) and all-cause mortality (RR 0.77, 95% CI 0.66–0.90, moderate-certainty evidence). However, supplementation has little effect on late-onset infection (RR 0.89, 95% CI 0.82–0.97, moderate-certainty evidence) [17]. A 2020 Network Meta-analysis (NMA) of 15,712 infants showed that *Lactobacillus* and *Bifidobacterium* combinations offered moderate-to-high-quality evidence for reducing all-cause mortality (OR 0.56, 95% CI 0.39–0.80, high-certainty evidence) and severe NEC (OR 0.35, 95% CI 0.20–0.59, high-certainty evidence) [28,34,35]. A 2023 NMA of 25,840 infants further established that only multi-strain formulations significantly reduced all-cause mortality compared with placebo (RR 0.69, 95% CI 0.56–0.86, high-certainty evidence) [36]. One probiotic is currently on the path to FDA approval, having received FDA Breakthrough Therapy designation: In the Connection Study, a phase 3 double-blind RCT enrolling 2158 VLBW infants across 95 NICUs, *Limosilactobacillus reuteri* IBP-9414 was safe and significantly reduced all-cause mortality (6.2% vs. 8.5%; RR 0.73, *p* = 0.036); however, neither primary endpoint—NEC incidence (RR 0.85, *p* > 0.05) nor time to sustained feeding tolerance (HR 1.10, *p* > 0.05)—reached statistical significance [37]. Key probiotic strains and representative clinical trial outcomes are summarized in Table 1 and the comparative efficacy of major probiotic formulations across outcomes is illustrated in Figure 2.

Despite these favorable pooled estimates, substantial heterogeneity across trials warrants careful interpretation. Probiotic strains differ considerably in their mechanisms, colonization efficiency, and clinical efficacy, and network meta-analyses find significant benefit for only a minority of studied strains, underscoring that probiotics cannot be treated as a uniform class. Formulation and manufacturing variability result in inconsistent colony-forming unit (CFU) counts at the time of administration. Feeding practices differ markedly across study populations, with human milk exposure, donor milk availability, and formula composition all influencing the gut microbiome. Baseline NEC incidence varies up to fivefold across participating NICUs, affecting achievable absolute risk reduction. Finally, optimal dose, timing, and duration of administration remain undefined. These sources of heterogeneity partially explain the variability in findings across individual trials and must be carefully weighed when translating pooled estimates to clinical decisions.

## 3. Prebiotics

Prebiotics are non-digestible nutrients that selectively stimulate the growth of beneficial intestinal microorganisms. Primary types studied in preterm infants include human milk oligosaccharides (HMOs), galacto-oligosaccharides (GOS), and fructo-oligosaccharides (FOS). Prebiotics promote commensal growth (*Bifidobacterium*, *Lactobacillus*) while limiting pathogens. Fermentation produces short-chain fatty acids (butyrate, propionate, acetate), which provide colonocyte energy, enhance epithelial maturation, and exert anti-inflammatory effects. They further modulate host immunity via gut-associated lymphoid tissue and pattern recognition receptors [18,40].

### Clinical Evidence in Preterm Infants

**Figure 3:**
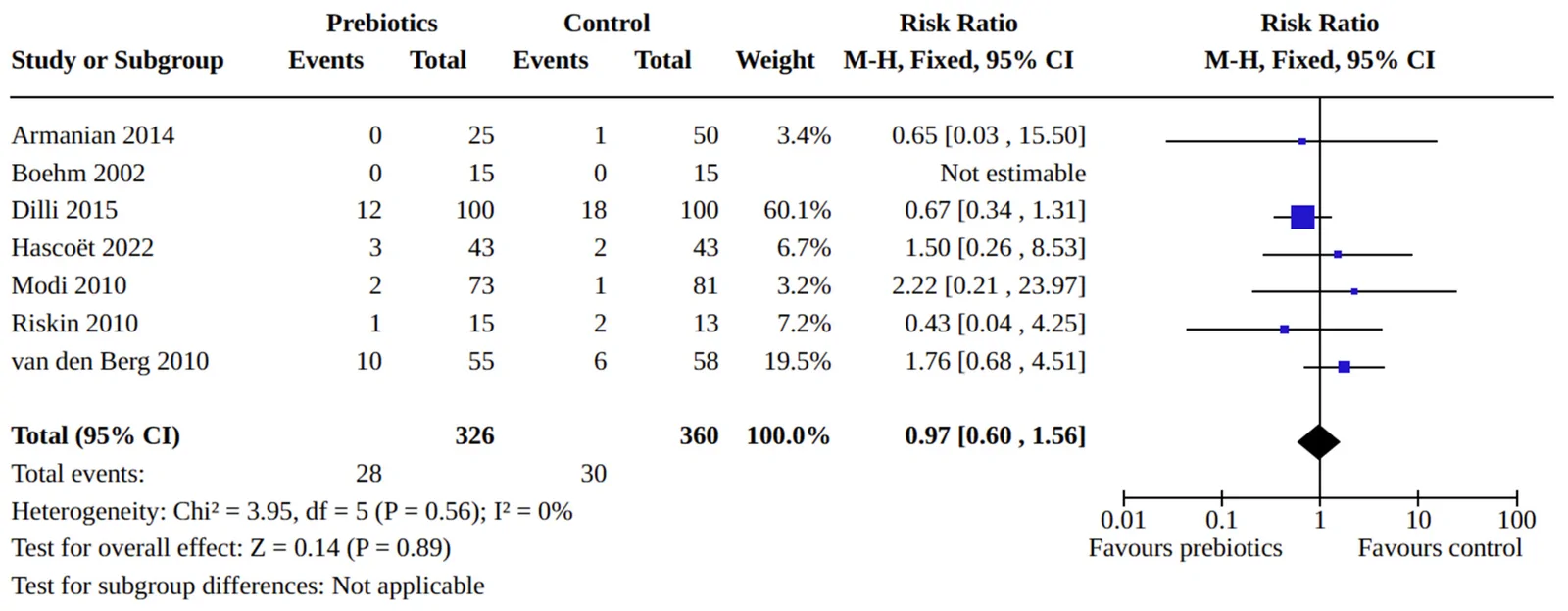
Forest plot showing the effect of prebiotic supplementation on NEC risk in preterm infants. Forest plot of randomized controlled trials included in the Cochrane Collaboration prebiotic review evaluating the effect of prebiotic supplementation compared with placebo/control on NEC incidence in preterm and very-low-birth-weight infants. Prebiotic supplementation did not demonstrate a statistically significant reduction in NEC risk compared with controls (RR 0.97, 95% CI 0.60–1.56). Adapted from a published Cochrane systematic review of prebiotics in preterm infants [36].

A 2023 Cochrane review (7 trials, 705 infants) found prebiotics may cause little or no difference in NEC (RR 0.97, 95% CI 0.60 to 1.56, low certainty of evidence), all-cause mortality (RR 0.43, 95% CI 0.20 to 0.92, low certainty of evidence), or late-onset infection (RR 0.79, 95% CI 0.60 to 1.06, low certainty of evidence) (Figure 3) [41].

Supplementing formula with prebiotics (90% GOS, 10% FOS) increases *Bifidobacterium* colonization and reduces pathogens compared to standard formula [42]. The current prebiotic evidence base is summarized in Figure 3. Small sample sizes and formulation heterogeneity render evidence insufficient for routine use in NEC prevention. Prebiotics do not have potential infection risks as probiotics but may be associated with reversible flatulence, bloating, and diarrhea [41].

## 4. Synbiotics

**Figure 4:**
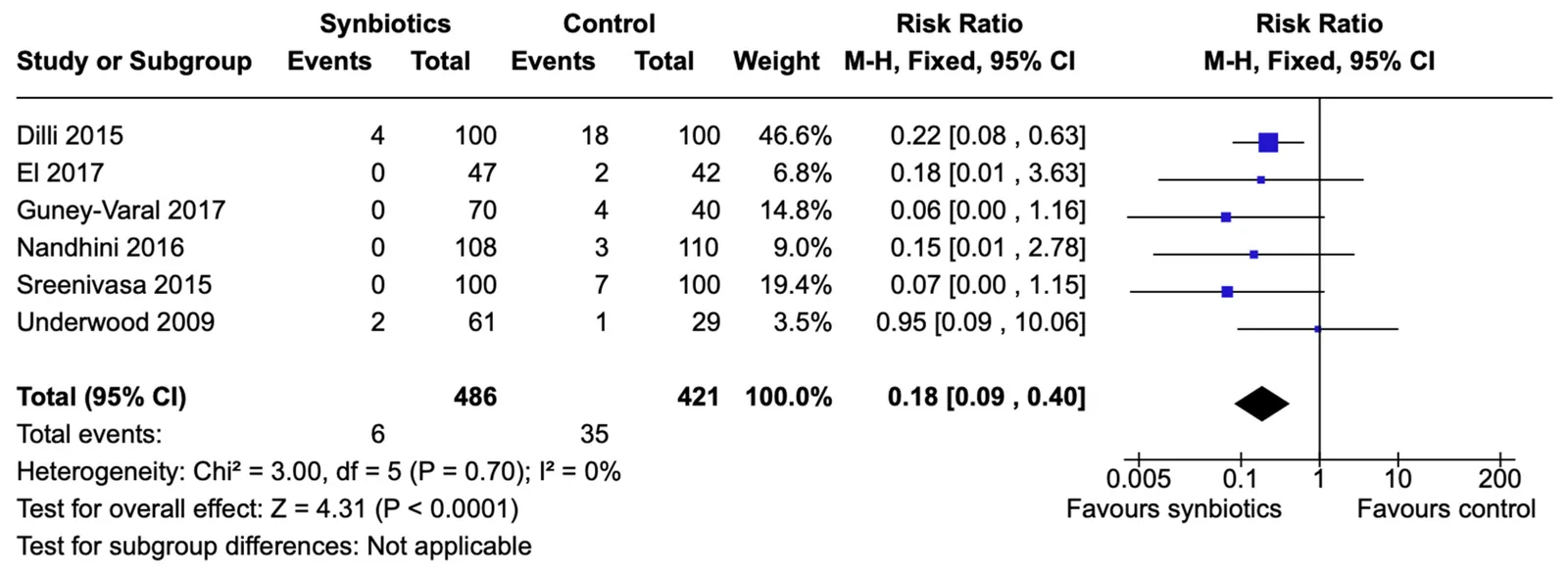
Forest plot showing the effect of synbiotic supplementation on NEC risk in preterm infants. Forest plot of randomized controlled trials included in the Cochrane Collaboration synbiotic review evaluating synbiotic supplementation for prevention of NEC in preterm and very-low-birth-weight infants. Overall, synbiotic supplementation was associated with a significant reduction in NEC incidence compared with placebo/control (RR 0.18, 95% CI 0.09–0.40). Adapted from a published Cochrane systematic review of synbiotics in preterm infants [38].

Synbiotics combine probiotics with selective prebiotic substrates to synergistically enhance microbial colonization and survival, potentially yielding health benefits superior to single-component therapies. A 2022 Cochrane review (6 trials, 925 infants) found synbiotics may reduce NEC (RR 0.18, 95% CI 0.09–0.40, low certainty of evidence) and mortality (RR 0.53, 95% CI 0.33–0.85, low certainty of evidence) [43]. A 2024 trial (118 VLBW infants) confirmed significant reductions in NEC (RR 0.22, 95% CI 0.07–0.72, low certainty of evidence) and feeding intolerance (RR 0.27, 95% CI 0.14–0.51, low certainty of evidence) [44]. The synbiotic evidence base and key trial outcomes are illustrated in Figure 4.

## 5. Comparative Effectiveness of Synbiotics vs. Probiotics

One network meta-analysis showed synbiotics associated with the lowest odds of NEC (OR 0.13, 95% CI 0.037–0.37) compared to other food supplements [45]. The 2023 JAMA Pediatrics network meta-analysis showed that multiple-strain probiotics plus oligosaccharides specifically outperformed probiotics alone for preventing severe NEC (RR 0.13, 95% CI 0.05–0.37 vs. RR 0.38, 95% CI 0.30–0.50) and reducing feeding intolerance [36]. The biological rationale for this superior efficacy rests on complementary mechanisms: prebiotic oligosaccharides enhance probiotic intestinal colonization and metabolic activity, while fermentation of these substrates produces short-chain fatty acids—particularly butyrate—that modulate epithelial integrity and exert anti-inflammatory effects relevant to NEC pathogenesis [43,46]. These synergistic mechanisms provide a coherent explanation for why combination approaches may outperform single-agent strategies, while highlighting that the prebiotic component must be chosen specifically to complement the probiotic strain used. However, evidence on the effectiveness and safety of synbiotics is currently limited by small cohorts and potential bias regarding allocation concealment and masking; large, high-quality trials are essential to inform definitive neonatal clinical policy [41,43].

## 6. Postbiotics

Postbiotics are “preparations of inanimate microorganisms and/or their components that confer health benefits”. Unlike probiotics, they utilize non-viable cells or metabolites instead of live organisms. Postbiotic types include short-chain fatty acids, lysates, extracellular vesicles, and peptides [47].

**Figure 5:**
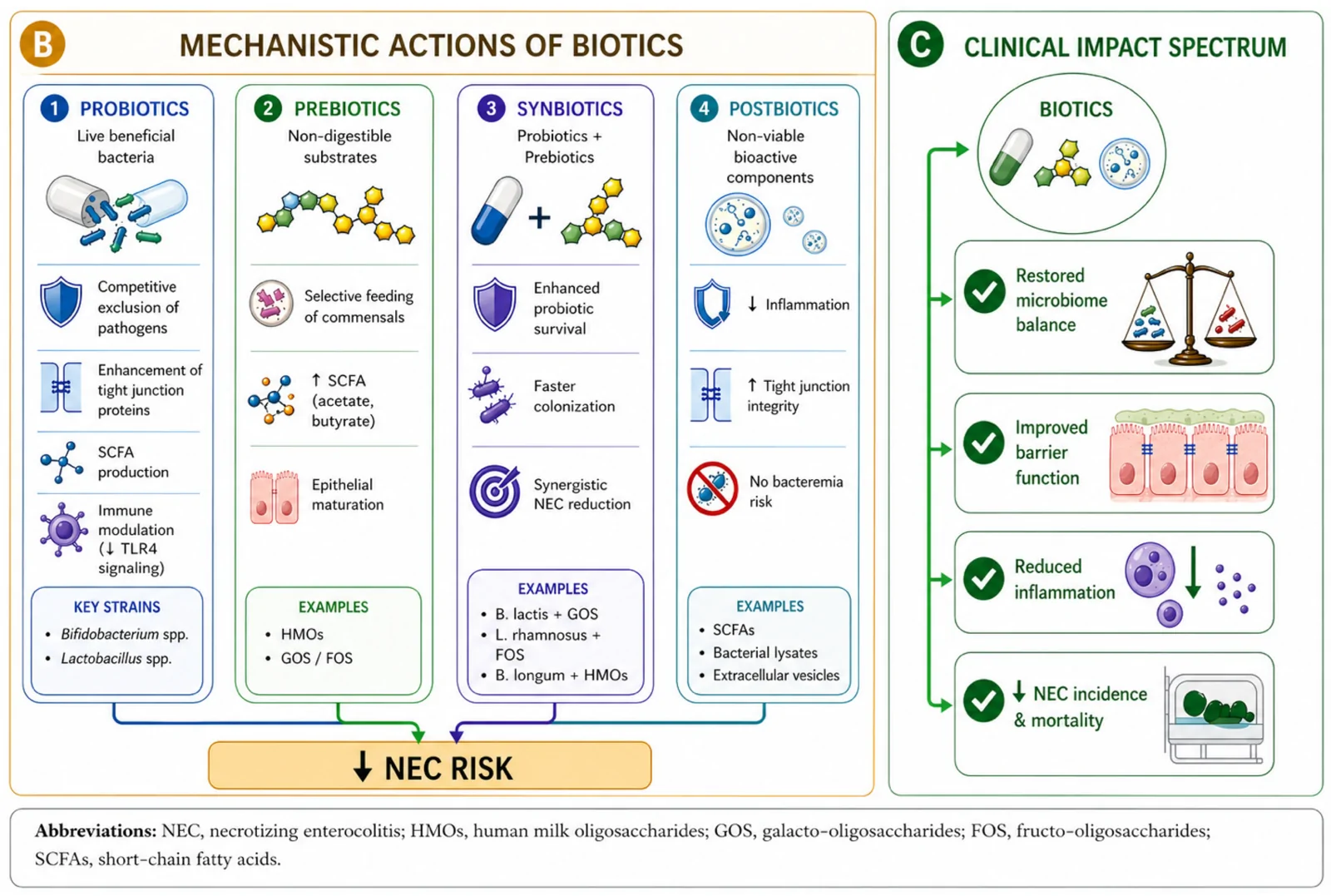
Mechanistic actions and clinical impact of biotics in NEC prevention. The figure summarizes the proposed mechanisms by which probiotics, prebiotics, synbiotics, and postbiotics may reduce NEC risk in preterm infants. These biotic therapies promote microbiome balance, enhance epithelial barrier integrity, increase short-chain fatty acid production, and reduce intestinal inflammation, ultimately contributing to lower NEC incidence and mortality. The figure was conceptually designed by the authors using artificial intelligence-assisted illustration support from ChatGPT Image 1 by OpenAI.

The mechanisms of action of postbiotics include modulating inflammatory pathways and attenuating immune responses such as Toll-like receptor 4 activation. Postbiotics strengthen the intestinal barrier by promoting tight junctions, mucus production, and maturation, which reduces translocation. Postbiotics may further shape the landscape of microbiota and induce protective responses via pattern recognition receptors [48]. Animal and in vitro gut models show postbiotics promote epithelial maturation and reduce inflammation [49]. An emerging and clinically relevant form of postbiotic is heat-inactivated probiotics, in which live probiotic bacteria are killed by controlled heat treatment, preserving their structural components and metabolic products while eliminating viability and the associated risk of sepsis. Early evidence in preterm neonates suggests that heat-inactivated preparations may retain anti-inflammatory and barrier-protective properties comparable to their live counterparts, potentially offering a safer alternative, particularly for ELBW infants, in whom the risk of bacteremia is highest [50]. However, these findings have not yet been validated in adequately powered neonatal clinical trials. To date, no large randomized controlled trial has established the efficacy, optimal dosing, or appropriate formulation of postbiotics for NEC prevention in preterm infants. Therefore, while postbiotics represent a scientifically promising and theoretically safer strategy—particularly for ELBW infants—they should currently be regarded as an experimental intervention requiring rigorous clinical investigation before routine use can be recommended. The mechanistic actions and clinical impact of biotics in NEC prevention are collectively summarized in Figure 5. 

## 7. Clinical Guidelines and Current Practice

### 7.1. American Academy of Pediatrics (AAP)

In 2021, the AAP clinical report stated that routine, universal administration of probiotics is not supported, particularly for infants <1000 g. This stance highlights the absence of FDA-regulated pharmaceutical-grade products in the U.S., where probiotics are sold as dietary supplements that bypass rigorous drug standards for safety, efficacy, and manufacturing. Despite this caution, the AAP acknowledged that trials involving over 10,000 infants and large meta-analyses demonstrate that multiple-strain probiotics significantly reduce NEC and all-cause mortality [19].

### 7.2. European Society for Paediatric Gastroenterology Hepatology and Nutrition (ESPGHAN)

In 2020, ESPGHAN provided conditional recommendations for specific formulations to reduce NEC: *L. rhamnosus* GG ATCC53103 (dose from 1 × 10^9^ to 6 × 10^9^ CFUs) or the combination of *B. infantis* Bb-02, *B. lactis* Bb-12, and *S. thermophilus* TH-4 (dose of 3.0 to 3.5 × 10^8^ CFUs of each strain). These recommendations are based on low certainty of evidence and require strict safety stipulations. ESPGHAN emphasized strain specificity, noting that their network meta-analysis found efficacy for only a minority of studied preparations [19,51].

### 7.3. World Health Organization (WHO)

In 2023, the WHO issued a conditional recommendation for probiotics in human-milk-fed infants born < 32 weeks’ gestation or <1.5 kg. This is supported by moderate-certainty evidence for reducing mortality and NEC [52].

### 7.4. American Gastroenterological Association (AGA)

The AGA’s 2020 guidelines conditionally suggest specific formulations—combinations of *Lactobacillus* and *Bifidobacterium*, *B. lactis*, *L. reuteri*, or *L. rhamnosus*—based on moderate-to-high-quality evidence [35].

### 7.5. Canadian Paediatric Society (CPS)

In 2019, the CPS reaffirmed the lack of safety and efficacy data for infants < 1000 g, recommending probiotics only for those >1 kg who are at risk of NEC [19].

## 8. Variability in NICU Adoption Worldwide

Probiotics adoption varies globally based on local regulations and institutional risk tolerance. While the AAP reports only 10% usage among extremely low gestational age U.S. neonates, a 2024 survey found 39% of NICUs now use probiotics, utilizing 14 different formulations. Common initiation criteria include birth weight < 1500 g and gestational age < 32 weeks, yet only 30% of units obtain formal parental consent. The 2021 AAP statement influenced decisions at 24% of hospitals—leading 11% to discontinue use—but 37% of non-users are now considering initiation. These conservative U.S. practices contrast with many European and Australian centers where routine administration is standard [19,53].

## 9. Controversies in the Field

### 9.1. Variability in Probiotic Preparations

Pooling studies that used diverse strains remain debated. Network meta-analyses find significant efficacy for only a minority of strains, highlighting the necessity of strain selection [51]. In addition, variability in CFU counts at manufacturing and after shelf storage results in inconsistent dosing, and the AGA technical review emphasized the need for studies defining optimal dose, timing, and frequency of administration [35]. ESPGHAN noted a lack of evidence for optimal start and length of probiotics treatment [51], while a 2024 survey identified 14 different formulations in U.S. NICUs [53]. Although current recommendations range from 1 × 10^8^ to 6 × 10^9^ CFUs depending on strain and formulation, dose–response relationships have not been systematically evaluated [19,51]. The PRIMAL trial administered fixed CFUs/strain over 28 days, yet the optimal duration remains unclear [54].

### 9.2. Regulatory Challenges

ESPGHAN recommended good manufacturing practices, local lab detection of bacteremia, and strains without antibiotic resistance genes. However, such quality control infrastructure is not universal. Persistent quality concerns, including contamination recalls and a 2023 FDA advisory regarding unapproved drug status, have led to sharp declines in U.S. probiotic administration [19,37,51,53,55]. A recent analysis examining probiotic use patterns and outcomes in preterm infants following the 2023 FDA warning action. That study documented a significant decline in probiotic administration rates across U.S. NICUs following the advisory, with subsequent trends in NEC incidence and mortality warranting close monitoring to assess the real-world impact of reduced probiotic exposure in this population. These findings underscore the importance of accelerating the development of regulated, pharmaceutical-grade probiotic products and of maintaining evidence-based guidance during periods of regulatory uncertainty [56].

### 9.3. Safety Concerns

Probiotic sepsis is rare, occurring in <0.5% of treated infants, primarily in ELBW neonates via translocation. Implicated species include *Bifidobacterium*, *Lactobacillus*, and *Saccharomyces*. A 2025 Canadian study reported sepsis rates of 1.4/1000 (<34 weeks) and 4/1000 (ELBW), with two deaths possibly linked to the intervention [57,58]. An AGA technical review referenced a case of fatal gastrointestinal mucormycosis from fungal contamination which underscores the need for stringent manufacturing oversight [35]. Population-level data strongly favor probiotics; meta-analyses identify them as a top intervention for reducing NEC and mortality. Protective mechanisms include pathogen exclusion, enhanced barrier function, and faster enteral feeding, which reduces intravenous access requirements. The safety profile remains reassuring, as quality issues appear manageable through rigorous control [57]. However, this reassuring overall picture must be balanced against the recognition that rare but serious adverse events do occur, including probiotic-associated sepsis and contamination-related fatalities. These events, although uncommon, are not trivial, particularly in ELBW infants in whom translocation risk is highest.

### 9.4. Biotics in Extremely Low Birth Weight (ELBW) Infants

The use of biotics in ELBW infants (<1000 g) represents one of the most clinically important and unresolved questions in neonatal medicine. This population carries the highest risk of NEC, yet paradoxically constitutes the group for which evidence is least robust and most conflicting. Several large randomized trials and their pre-specified subgroup analyses have failed to demonstrate consistent benefit in ELBW infants. The PiPS trial found no reduction in NEC, sepsis, or mortality with B. breve [33]. The ProPrems trial, despite showing overall NEC reduction, found no benefit in infants born at less than 28 weeks’ gestation or with birth weight under 1000 g [32]. Similarly, the 2023 Cochrane review and major network meta-analyses note that while multi-strain probiotics reduce NEC in VLBW infants broadly, the certainty of evidence specifically in ELBW infants remains low, partly due to the small absolute numbers of ELBW infants recruited in trials [17]. Safety considerations are also heightened in this group, with probiotic-associated sepsis occurring at higher rates (approximately 4/1000) in ELBW compared to less premature infants. Both the AAP and the CPS specifically advise against routine probiotic use in infants under 1000 g [19]. Future research must prioritize adequately powered, ELBW-focused pragmatic trials that define optimal strain selection, dose, timing, and duration within this high-risk population, and should report ELBW subgroup outcomes as a primary, rather than secondary, endpoint.

### 9.5. Generalizability of Evidence

International NEC rates vary substantially, with a fourfold variation among infants < 32 weeks (2–7%) and fivefold among those <1000 g (5–22%) across high-income countries [59]. A 2025 study identified low socioeconomic status as an independent risk factor [60]. Feeding type influences probiotic efficacy. Human milk reduces risks of NEC, sepsis, and neurodevelopmental impairment [61]. A 2024 Cochrane review demonstrated that donor human milk reduces the risk of NEC by about half (RR 0.53, 95% CI 0.37–0.76; high-certainty evidence) compared to formula in very preterm or VLBW infants [62]. While the ProPrems trial involved a population with high human milk exposure, robust donor milk programs in some centers already minimize baseline NEC rates, which may limit the incremental benefit of probiotic supplementation [19,35]. A mother’s own milk provides a uniquely tailored combination of HMOs, immunoglobulins, lactoferrin, and bioactive factors that collectively support a protective microbiome [63,64]. Donor human milk, while superior to formula, has a different HMO profile than a mother’s own milk and may therefore confer a different modulatory effect on probiotic colonization [65]. These interactions may partially explain differences in probiotic efficacy observed across studies and NICU populations, and underscore the importance of considering feeding type as a key co-variable in future biotic trials.

### 9.6. Cross-Colonization in the NICU Environment

Environmental uptake is an important clinical trial consideration. In the PRIMAL trial, probiotic bacteria were detected in 49% of placebo infants, particularly among siblings of treated infants, suggesting low interindividual microbiome barriers, especially for *Bifidobacterium* infantis, which may dilute measured treatment effects [54]. Similarly, in the PiPS trial, substantial cross-colonization was observed in the control arm: *B. breve* BBG-001 was detected in the stools of 49% of placebo-allocated infants by 36 weeks postmenstrual age [33].

### 9.7. Antibiotic Exposure and Probiotic Efficacy

Antibiotics disrupt preterm microbiota, influencing efficacy [19]. Recent research indicates probiotics significantly reduce antibiotic resistance gene prevalence and multidrug-resistant pathogen loads. Despite this, persistent MDR Enterococcus and its high horizontal gene transfer potential require ongoing surveillance and further research into probiotics as tools for antimicrobial stewardship [66].

### 9.8. Ethical Considerations

#### 9.8.1. Equipoise for Further Trials

Efficacy gaps in extremely preterm infants and regulatory concerns maintain genuine equipoise for further placebo-controlled trials. The 2023 Cochrane review highlighted the need for large, high-quality trials to inform clinicians and policy. Feasible models like the PRIMAL trial provide vital mechanistic insights into environmental dynamics, remaining essential for resolving current clinical uncertainties [17,54].

#### 9.8.2. Informed Consent and Parental Communication

The ESPGHAN panel stipulated that the potential risks and benefits must be provided to parents of preterm infants [19,51]. This fulfills ethical obligations for interventions lacking pharmaceutical-grade approval. The PRIMAL trial demonstrated that formal consent processes are feasible [54]. However, optimal communication and consent strategies require further guidance as probiotic adoption increases.

## 10. Future Directions

### Precision Microbiome Approaches

Personalized strategies shift universal supplementation toward individualized interventions tailored to an infant’s unique microbial profile and risk factors. For instance, *B. infantis* requires HMOs to achieve gut dominance and reduce antibiotic resistance. These findings highlight the need to consider interactions among probiotic strain characteristics, diet composition, and host factors when designing personalized interventions [67,68,69]. Longitudinal profiling reveals signatures preceding NEC, offering early detection via biomarkers like *C. neonatale* and *C. perfringens*. Pre-onset fecal samples demonstrate high *C. perfringens* abundance and reduced alpha diversity. Targeted modulation could facilitate earlier diagnosis and more effective prevention [12,22,70].

Metagenomic sequencing identifies specific *E. coli* subtypes as NEC risk factors [70]. Shotgun analyses reveal opportunistic bacterial overgrowth and reduced diversity in affected infants. Multi-kingdom profiling using 16S and shotgun methods provides deep insights into microbial function, virulence, and resistance, helping delineate host–microbe interactions in NEC pathogenesis. Analyses have identified DL-lactate accumulation as a pre-NEC biomarker. Serum profiling shows NEC patients have significantly lower amino acid levels (e.g., ornithine, arginine), which correlate with beneficial microbes. Volatile organic compound (VOC) signatures distinguish NEC with high accuracy (AUC = 0.82). Additionally, short-chain fatty acids like acetate and butyrate modulate immunity and barrier function through epigenetic mechanisms [12,22,70]. Integrating multi-omics with machine learning algorithms enables predictive models to identify high-risk infants before clinical onset [12,71]. While fecal microbiota and VOC signatures are promising early biomarkers, larger, diverse validation cohorts are necessary before clinical implementation [70].

Beyond microbiome composition, future precision-prevention strategies must integrate a broader panel of biomarkers reflecting intestinal inflammation, epithelial injury, and host–microbiome interactions. Circulating markers of intestinal inflammation—such as fecal calprotectin, serum intestinal fatty acid binding protein (I-FABP), and urinary claudin-3—reflect epithelial injury and barrier disruption that precede clinical NEC. Among inflammatory cytokines, IL-6, IL-8, and IL-10 show the most robust serum elevations in NEC. Integrating these host-derived biomarkers with microbiome profiling through similar computational and multi-omics frameworks may improve risk stratification, facilitate earlier diagnosis, and enable individualized targeting of biotic interventions to infants most likely to benefit [72,73,74]. An emerging area of investigation involves the potential role of selenium deficiency in NEC pathogenesis. Gürünlüoğlu et al. (2025) conducted transcriptomic profiling in premature neonates with NEC and identified significant downregulation of multiple selenoprotein-encoding genes, alongside upregulation of genes involved in hypoxia-induced apoptosis and inflammation [75]. Selenoproteins serve critical antioxidant functions in the intestinal epithelium, and selenium deficiency activates NF-κB signaling, induces oxidative stress, and disrupts intestinal barrier integrity—mechanisms that converge with established NEC pathways, suggesting selenium deficiency may be a previously underrecognized contributor [76]. Prospective studies evaluating selenium status prior to NEC onset are needed to establish causality.

Despite substantial progress in understanding the microbiome’s role in NEC, critical research gaps remain. Research must prioritize strains supported by moderate-to-high-certainty evidence [17,34]. Large, blinded trials are urgently needed for ELBW infants—the population at greatest risk yet most underrepresented in existing trials—to define optimal dosing, timing, and duration relative to the infant’s developmental window [17]. Because many strains remain unstudied, probiotics cannot be recommended as a uniform class; functional and genomic research should guide future candidate selection [68]. Developing FDA-regulated, pharmaceutical-grade probiotics is a critical priority. These products must meet drug standards for strain identity, shelf-life viability, and safety—criteria no existing trial has yet satisfied. Global regulatory harmonization is essential to standardize identification, stability assessment, and contamination screening throughout the full product lifecycle [19,51,55].

Postbiotics offer safety advantages by eliminating infection risks in extremely preterm infants enhancing gut maturation and exerting anti-inflammatory effects. Despite promising preclinical data, clinical trials must still define optimal dosing and formulations for postbiotics [70,77]. Synthetic microbial communities—engineered consortia of human-derived gut bacteria—offer a standardized and customizable alternative to conventional probiotics. These consortia mimic native microbiota functions and provide colonization resistance. While both consortium and fecal transplants protect NEC mouse models, probiotic consortium transplantation (PCT) is preferable for pediatrics due to its controllable, reproducible composition [68,78]. Microbiome engineering shapes early gut colonization to foster NEC resilience, utilizing multi-kingdom profiling to enable rational intervention design [67].

Several additional technologies show early promise but remain experimental. Biofilm-based delivery systems outperform planktonic probiotics. In piglet models, *L. reuteri* grown on dextranomer microspheres significantly reduces NEC, mortality, and neuroinflammation [79]. Flash nanoencapsulation (FNE) improves probiotic adhesion and environmental resistance. In NEC models, encapsulated L. rhamnosus GG reduces inflammation via TLR4–NFκB modulation and strengthens epithelial barrier integrity [80]. Virus-like particles (30 kDa–0.45 µm) reduce NEC severity in preterm piglets by eliminating pathogens. This virome transfer diversifies viral communities and modulates bacterial populations, highlighting the role of maintaining intestinal homeostasis [81]. Because therapeutic efficacy depends on the specific disease state, further investigation is required before clinical use. Rapidly evolving neonatal virome sequencing will inform future strategies for phage selection, dosing, and resistance prevention [68,82,83,84].

It is important to emphasize the distinction between interventions already supported by translational and observational clinical data—such as precision microbiome profiling, biomarker-guided patient selection, and probiotic timing optimization—and those that remain primarily preclinical, including synthetic microbial communities, nanoencapsulation platforms, bacteriophage therapy, and fecal virome transfer. The latter group, while scientifically promising, has not yet been evaluated in adequately powered neonatal clinical trials; safety, efficacy, and optimal formulation remain undefined. These technologies should be regarded as experimental, and clinical implementation cannot be recommended outside carefully designed trial frameworks. Future research must proceed in a stepwise fashion, with rigorous safety evaluation preceding any broader application in this vulnerable population.

## 11. Summary

Evidence suggests probiotics reduce NEC and mortality in VLBW infants, though certainty remains low to moderate. Network meta-analyses indicate multi-strain *Lactobacillus* and *Bifidobacterium* formulations are most effective, as only multi-strain products significantly reduced all-cause mortality. Synbiotics are promising for NEC and mortality; specifically, multi-strain probiotics plus oligosaccharides are superior for severe NEC compared to probiotics alone. Conversely, prebiotics alone show little effect, and postbiotics require further clinical evaluation. A critical evidence gap remains for ELBW infants, where probiotics show no clear benefit, and large trials failed to demonstrate efficacy in this subgroup probably because of low numbers of ELBW infants recruited [17,19,35,36,41,43,78]. Conflicting professional guidance persists; the AAP and CPS advise against routine use in infants < 1000 g. Decision-making should involve formalized parental consent and meticulous outcome documentation, given the off-label status and lack of FDA approval. Clinicians must evaluate probiotics within existing frameworks, as robust human milk programs may limit incremental benefits [19,51,55,68,69].

Future NEC prevention will rely on precision approaches integrating multi-omics and individualized therapy. [12,22,70,77]. Next-generation probiotics utilizing biofilm delivery or nanoencapsulation may reduce required dosing while enhancing therapeutic impact [35,80]. Bacteriophage therapy and fecal virome transfer provide specific modulation without disrupting commensals [81]. Synthetic communities and probiotic consortia offer standardized, customizable bacterial supplementation tailored to individual infants. Further advancement requires pharmaceutical-grade products, ELBW-focused pragmatic trials, and international regulatory harmonization. Integrating machine learning with multi-omics may identify high-risk infants and guide personalized interventions. Only through coordinated, evidence-based advancement can microbiome-targeted therapies truly reduce the burden of NEC in vulnerable preterm infants.

## Figures and Tables

**Table 1:** Strain-specific effects.

Probiotic Strain	Evidence Base	Population	Key Outcomes	Effect Summary	Certainty of Evidence	Ref.
***B. breve* (BBG-001)**	PiPS RCT	Very preterm (*n* = 1315)	NEC, sepsis, mortality	No effect on NEC (RR 0.93), sepsis (RR 0.97), or mortality (RR 0.93)	Moderate to High (AGA)	[19,33,34]
***B. infantis + S. thermophilus + B. lactis***	ProPrems RCT	VLBW (*n* = 1099)	NEC	↓ NEC (2.0% vs. 4.4%; RR 0.46); no benefit <28 wk or <1000 g	Low (AGA)	[19,32,34]
***L. rhamnosus* GG**	SR/MA (5 RCT)	Preterm (*n* = 851)	NEC ≥ II, severe NEC	↓ NEC ≥ II (RR 0.50)	Low (ESPGHAN)	[38]
	NMA	Preterm (*n* = 15,712)	Severe NEC	↓ severe NEC (OR 0.44)	Moderate	[34]
***L. reuteri***	SR (12 RCT)	Preterm (*n* = 2284)	NEC, LOS, feeding	↓ NEC (RR 0.57); ↓ LOS (RR 0.72); faster feeds (−2.7 days)	Very Low	[39]
	Connection RCT (Phase 3)	VLBW (*n* = 2117)	NEC, Sustained feeding tolerance (SFT), Mortality	↓ Mortality (6.2% vs. 8.5%, RR 0.73); no significant effect on NEC (RR 0.85) or SFT (HR 1.10)		[37]
***B. animalis* subsp. *lactis***	NMA	Preterm/ VLBW	Severe NEC, Hospital Stay	↓ severe NEC (OR 0.31); ↓ Stay (−13.0 days)	Moderate to High	[34]
***S. boulardii* (combinations)**	NMA	Preterm	Feeding	Faster feeds (−3.3 days)	Moderate to High	[34,35]
***Lactobacillus* + *Bifidobacterium* spp.**	NMA	Large pooled Preterm cohorts	Mortality, Severe NEC	↓ Mortality (OR 0.56); ↓ Severe NEC (OR 0.35)	Moderate to High	[34,35]

**Abbreviations:** NEC, necrotizing enterocolitis; VLBW, very-low-birth-weight; RCT, randomized controlled trial; RR, risk ratio; OR, odds ratio; SR/MA, systematic review and meta-analysis; NMA, network meta-analysis; LOS, late-onset sepsis; AGA, American Gastroenterological Association; ESPGHAN, European Society for Paediatric Gastroenterology Hepatology and Nutrition; ↓ decrease.

## Data Availability

No new data were created or analyzed in this study. Data sharing is not applicable to this article.

## References

[1] Neu J., Walker W.A. (2011). Necrotizing enterocolitis. N. Engl. J. Med..

[2] Fitzgibbons S.C., Ching Y., Yu D., Carpenter J., Kenny M., Weldon C., Lillehei C., Valim C., Horbar J.D., Jaksic T. (2009). Mortality of necrotizing enterocolitis expressed by birth weight categories. J. Pediatr. Surg..

[3] Schulzke S.M., Deshpande G.C., Patole S.K. (2007). Neurodevelopmental outcomes of very low-birth-weight infants with necrotizing enterocolitis: A systematic review of observational studies. Arch. Pediatr. Adolesc. Med..

[4] Han S.M., Knell J., Henry O., Riley H., Hong C.R., Staffa S.J., Modi B.P., Jaksic T. (2020). Long-term outcomes of severe surgical necrotizing enterocolitis. J. Pediatr. Surg..

[5] Nino D.F., Sodhi C.P., Hackam D.J. (2016). Necrotizing enterocolitis: New insights into pathogenesis and mechanisms. Nat. Rev. Gastroenterol. Hepatol..

[6] Li B., Yeganeh M., Lee D., Chusilp S., Balsamo F., Ganji N., Wang C.-Y., Zito A., Biouss G., Pierro A. (2026). Exploring the Complex Pathophysiology of Necrotizing Enterocolitis in Preterm Neonates. Annu. Rev. Pathol..

[7] Hackam D.J., Sodhi C.P. (2022). Bench to bedside—New insights into the pathogenesis of necrotizing enterocolitis. Nat. Rev. Gastroenterol. Hepatol..

[8] Farghaly M.A.A., Alzayyat S., Kassim D., Taha S.A., Aly H., Mohamed M.A. (2024). Maternal chorioamnionitis and the risk for necrotizing enterocolitis in the United States: A national cohort study. Early Hum. Dev..

[9] Garg P.M., Riddick R., Ansari M.A.Y., Rebentisch A., Shetty A., Adams K., Hillegass W., Garg P.P. (2026). Association of Placental Pathology and Antibiotic Exposure after Birth with the Severity of Necrotizing Enterocolitis in Preterm Infants: A Case-Control Study. Am. J. Perinatol..

[10] March M.I., Gupta M., Modest A.M., Wu L., Hacker M.R., Martin C.R., Rana S. (2015). Maternal risk factors for neonatal necrotizing enterocolitis. J. Matern.-Fetal Neonatal Med..

[11] Pammi M., Cope J., Tarr P.I., Warner B.B., Morrow A.L., Mai V., Gregory K.E., Kroll J.S., McMurtry V., Ferris M.J. (2017). Intestinal dysbiosis in preterm infants preceding necrotizing enterocolitis: A systematic review and meta-analysis. Microbiome.

[12] Thänert R., Keen E.C., Dantas G., Warner B.B., Tarr P.I. (2021). Necrotizing Enterocolitis and the Microbiome: Current Status and Future Directions. J. Infect. Dis..

[13] Blum L., Vincent D., Boettcher M., Knopf J. (2024). Immunological aspects of necrotizing enterocolitis models: A review. Front. Immunol..

[14] Nanthakumar N., Meng D., Goldstein A.M., Zhu W., Lu L., Uauy R., Llanos A., Claud E.C., Walker W.A. (2011). The mechanism of excessive intestinal inflammation in necrotizing enterocolitis: An immature innate immune response. PLoS ONE.

[15] Zhou Y., Li Y., Zhou B., Chen K., Lyv Z., Huang D., Liu B., Xu Z., Xiang B., Jin S. (2017). Inflammation and Apoptosis: Dual Mediator Role for Toll-like Receptor 4 in the Development of Necrotizing Enterocolitis. Inflamm. Bowel Dis..

[16] Hackam D.J., Afrazi A., Good M., Sodhi C.P. (2013). Innate immune signaling in the pathogenesis of necrotizing enterocolitis. Clin. Dev. Immunol..

[17] Sharif S., Meader N., Oddie S.J., Rojas-Reyes M.X., McGuire W. (2023). Probiotics to prevent necrotising enterocolitis in very preterm or very low birth weight infants. Cochrane Database Syst. Rev..

[18] Weingarden A.R., Ko C.W. (2024). Non-prescription Therapeutics. Am. J. Gastroenterol..

[19] Poindexter B., Cummings J., Hand I., Adams-Chapman I., Aucott S.W., Puopolo K.M., Goldsmith J.P., Kaufman D., Martin C., Committee on Fetus and Newborn (2021). Use of Probiotics in Preterm Infants. Pediatrics.

[20] La Rosa P.S., Warner B.B., Zhou Y., Weinstock G.M., Sodergren E., Hall-Moore C.M., Stevens H.J., Bennett W.E., Shaikh N., Linneman L.A. (2014). Patterned progression of bacterial populations in the premature infant gut. Proc. Natl. Acad. Sci. USA.

[21] Warner B.B., Deych E., Zhou Y., Hall-Moore C., Weinstock G.M., Sodergren E., Shaikh N., Hoffmann J.A., Linneman L.A., Hamvas A. (2016). Gut bacteria dysbiosis and necrotising enterocolitis in very low birthweight infants: A prospective case-control study. Lancet.

[22] Tam P.K.H., Chung P.H.Y., St Peter S.D., Gayer C.P., Ford H.R., Tam G.C.H., Wong K.K.Y., Pakarinen M.P., Davenport M. (2017). Advances in paediatric gastroenterology. Lancet.

[23] Chae H., Kim S.Y., Kang H.M., Im S.A., Youn Y.A. (2024). Dysbiosis of the initial stool microbiota increases the risk of developing necrotizing enterocolitis or feeding intolerance in newborns. Sci. Rep..

[24] Onofre M.J., Augusto Cirino Silva C., Caixeta da Silveira Ferreira I., Von Dolinger de Brito Röder D. (2025). Gut microbiota as a risk and protective factor in neonatal necrotizing enterocolitis: An integrative review. Early Hum. Dev..

[25] Qiu J., Wu S., Huang R., Liao Z., Pan X., Zhao K., Peng Y., Xiang S., Cao Y., Ma Y. (2024). Effects of antibiotic therapy on the early development of gut microbiota and butyrate-producers in early infants. Front. Microbiol..

[26] Nguyen T., Bürkin F., Ayala-Montaño S., Monterrosa I.A., Jonas D., Klotz D., Fuchs H., Kuntz M., Schneider C., Wolkewitz M. (2025). Prospective Whole-Genome Sequencing to Identify Bacterial Transmission and Its Modifiers in Neonates. JAMA Netw. Open.

[27] Lai C., Huang L., Wang Y., Huang C., Luo Y., Qin X., Zeng J. (2024). Effect of different delivery modes on intestinal microbiota and immune function of neonates. Sci. Rep..

[28] Su G.L., Ko C.W., Bercik P., Falck-Ytter Y., Sultan S., Weizman A.V., Morgan R.L. (2020). AGA Clinical Practice Guidelines on the Role of Probiotics in the Management of Gastrointestinal Disorders. Gastroenterology.

[29] Bin-Nun A., Bromiker R., Wilschanski M., Kaplan M., Rudensky B., Caplan M., Hammerman C. (2005). Oral probiotics prevent necrotizing enterocolitis in very low birth weight neonates. J. Pediatr..

[30] Dani C., Biadaioli R., Bertini G., Martelli E., Rubaltelli F.F. (2002). Probiotics feeding in prevention of urinary tract infection, bacterial sepsis and necrotizing enterocolitis in preterm infants. A prospective double-blind study. Biol. Neonate.

[31] Lin H.C., Su B.H., Chen A.C., Lin T.W., Tsai C.H., Yeh T.F., Oh W. (2005). Oral probiotics reduce the incidence and severity of necrotizing enterocolitis in very low birth weight infants. Pediatrics.

[32] Jacobs S.E., Tobin J.M., Opie G.F., Donath S., Tabrizi S.N., Pirotta M., Morley C.J., Garland S.M. (2013). Probiotic effects on late-onset sepsis in very preterm infants: A randomized controlled trial. Pediatrics.

[33] Costeloe K., Bowler U., Brocklehurst P., Hardy P., Heal P., Juszczak E., King A., Panton N., Stacey F., Whiley A. (2016). A randomised controlled trial of the probiotic Bifidobacterium breve BBG-001 in preterm babies to prevent sepsis, necrotising enterocolitis and death: The Probiotics in Preterm infantS (PiPS) trial. Health Technol. Assess..

[34] Morgan R.L., Preidis G.A., Kashyap P.C., Weizman A.V., Sadeghirad B., McMaster Probiotic, Prebiotic, and Synbiotic Work Group (2020). Probiotics Reduce Mortality and Morbidity in Preterm, Low-Birth-Weight Infants: A Systematic Review and Network Meta-analysis of Randomized Trials. Gastroenterology.

[35] Preidis G.A., Weizman A.V., Kashyap P.C., Morgan R.L. (2020). AGA Technical Review on the Role of Probiotics in the Management of Gastrointestinal Disorders. Gastroenterology.

[36] Wang Y., Florez I.D., Morgan R.L., Foroutan F., Chang Y., Crandon H.N., Zeraatkar D., Bala M.M., Mao R.Q., Tao B. (2023). Probiotics, Prebiotics, Lactoferrin, and Combination Products for Prevention of Mortality and Morbidity in Preterm Infants: A Systematic Review and Network Meta-Analysis. JAMA Pediatr..

[37] Neu J., Del Moral T., Guthrie S.O., Hudak M.L., Indrio F., Kim J.H., Kronström A., Martin C.R., Modi N., Rastad J. (2026). Live biotherapeutic product IBP-9414 (*L. reuteri*) in very low birth weight infants: The Connection Study. Pediatr. Res..

[38] Ananthan A., Balasubramanian H., Rath C., Muthusamy S., Rao S., Patole S. (2024). Lactobacillus rhamnosus GG as a probiotic for preterm infants: A strain specific systematic review and meta-analysis. Eur. J. Clin. Nutr..

[39] Ang J.L., Athalye-Jape G., Rao S., Bulsara M., Patole S. (2023). Limosilactobacillus reuteri DSM 17938 as a probiotic in preterm infants: An updated systematic review with meta-analysis and trial sequential analysis. J. Parenter. Enter. Nutr..

[40] Yalçıntaş Y.M., Bolino M.J., Duman H., Sarıtaş S., Pekdemir B., Kalkan A.E., Canbolat A.A., Bolat E., Eker F., Rocha J.M. (2025). Prebiotics: Types, selectivity and utilization by gut microbes. Int. J. Food Sci. Nutr..

[41] Sharif S., Oddie S.J., Heath P.T., McGuire W. (2023). Prebiotics to prevent necrotising enterocolitis in very preterm or very low birth weight infants. Cochrane Database Syst. Rev..

[42] Borewicz K., Suarez-Diez M., Hechler C., Beijers R., de Weerth C., Arts I., Penders J., Thijs C., Nauta A., Lindner C. (2019). The effect of prebiotic fortified infant formulas on microbiota composition and dynamics in early life. Sci. Rep..

[43] Sharif S., Heath P.T., Oddie S.J., McGuire W. (2022). Synbiotics to prevent necrotising enterocolitis in very preterm or very low birth weight infants. Cochrane Database Syst. Rev..

[44] Mahboobipour A.A., Bitaraf A., Mohammadi P., Khosravifar M., Babaei H., Shahidolahi A. (2024). Effects of synbiotics on necrotizing enterocolitis and full enteral feeding in very low birth weight infants: A double-blind, randomized controlled trial. Medicine.

[45] Zhou J., Yang M., Wang F., Liu S., Hei M., Jiang M. (2023). Assessment of food supplements for the prevention of necrotizing enterocolitis in preterm neonates: A systematic review and network meta-analysis. Comput. Biol. Med..

[46] Yu W., Venkatraman A., Menden H.L., Martinez M., Umar S., Sampath V. (2023). Short-chain fatty acids ameliorate necrotizing enterocolitis-like intestinal injury through enhancing Notch1-mediated single immunoglobulin interleukin-1-related receptor, toll-interacting protein, and A20 induction. Am. J. Physiol.-Gastrointest. Liver Physiol..

[47] Gibson G.R., Hutkins R., Sanders M.E., Prescott S.L., Reimer R.A., Salminen S.J., Scott K., Stanton C., Swanson K.S., Cani P.D. (2017). Expert consensus document: The International Scientific Association for Probiotics and Prebiotics (ISAPP) consensus statement on the definition and scope of prebiotics. Nat. Rev. Gastroenterol. Hepatol..

[48] Gianni M.L., Morniroli D., Mosca F., Rescigno M. (2024). Can Postbiotics Represent a New Strategy for NEC?. Adv. Exp. Med. Biol..

[49] Zheng N., Gao Y., Zhu W., Meng D., Walker W.A. (2020). Short chain fatty acids produced by colonizing intestinal commensal bacterial interaction with expressed breast milk are anti-inflammatory in human immature enterocytes. PLoS ONE.

[50] Athalye-Jape G., Esvaran M., Patole S., Nathan E.A., Doherty D.A., Sim E., Chandrasekaran L., Kok C., Schuster S., Conway P. (2025). Effects of a live versus heat-inactivated probiotic Bifidobacterium spp in preterm infants: A randomised clinical trial. Arch. Dis. Child.-Fetal Neonatal Ed..

[51] van den Akker C.H.P., van Goudoever J.B., Shamir R., Domellof M., Embleton N.D., Hojsak I., Lapillonne A., Mihatsch W.A., Canani R.B., Bronsky J. (2020). Probiotics and Preterm Infants: A Position Paper by the European Society for Paediatric Gastroenterology Hepatology and Nutrition Committee on Nutrition and the European Society for Paediatric Gastroenterology Hepatology and Nutrition Working Group for Probiotics and Prebiotics. J. Pediatr. Gastroenterol. Nutr..

[52] Care of Preterm or Low Birthweight Infants Group (2023). New World Health Organization recommendations for care of preterm or low birth weight infants: Health policy. EClinicalMedicine.

[53] Hanna M., Ahmad I., Yanowitz T., Kim J., Hunter C., DiGeronimo R., Ahmad K.A., Sullivan K., Markel T.A., Hair A.B. (2024). Current Patterns of Probiotic Use in U.S. Neonatal Intensive Care Units: A Multi-Institution Survey. Am. J. Perinatol..

[54] Van Rossum T., Haiß A., Knoll R.L., Marißen J., Podlesny D., Pagel J., Bleskina M., Vens M., Fortmann I., Siller B. (2024). Bifidobacterium and Lactobacillus Probiotics and Gut Dysbiosis in Preterm Infants: The PRIMAL Randomized Clinical Trial. JAMA Pediatr..

[55] Viswanathan S., Gautham K.S. (2026). Navigating the United States FDA advisory: Probiotics in Preterm Infants. J. Perinatol..

[56] Tolia V.N., Bennett M.M., Handler D., Canvasser J., Greenberg R.G., Ursprung R., Ahmad K.A., Patel R.M. (2026). Probiotics and necrotizing enterocolitis in preterm infants after the food and drug administration warning actions. J. Perinatol..

[57] Embleton N.D., van den Akker C.H.P., Alshaikh B.N. (2025). Probiotic supplementation—Does it prevent or cause neonatal sepsis?. Semin. Fetal Neonatal Med..

[58] Younge N., Patel R.M. (2025). Probiotics and the Risk of Infection. Clin. Perinatol..

[59] Battersby C., Santhalingam T., Costeloe K., Modi N. (2018). Incidence of neonatal necrotising enterocolitis in high-income countries: A systematic review. Arch. Dis. Child.-Fetal Neonatal Ed..

[60] Nasher O., Wayne C.D., Cen R., Mills E., Van Arendonk K.J., Besner G.E. (2026). Association of socioeconomic status with development and severity of necrotizing enterocolitis. J. Pediatr. Surg..

[61] Parker M.G., Stellwagen L., Miller E.R., Noble L., Corkins M.R., Hudak M.L., Eichenwald E., Ambalavanan N., Guillory C., Kaufman D. (2026). Promoting Human Milk and Breastfeeding for the Very Low Birth Weight Infant: Clinical Report. Pediatrics.

[62] Quigley M., Embleton N.D., Meader N., McGuire W. (2024). Donor human milk for preventing necrotising enterocolitis in very preterm or very low-birthweight infants. Cochrane Database Syst. Rev..

[63] Reniker L.N., Frazer L.C., Good M. (2023). Key biologically active components of breast milk and their beneficial effects. Semin. Pediatr. Surg..

[64] Verhasselt V., Tellier J., Carsetti R., Tepekule B. (2024). Antibodies in breast milk: Pro-bodies designed for healthy newborn development. Immunol. Rev..

[65] Marx C., Bridge R., Wolf A.K., Rich W., Kim J.H., Bode L. (2014). Human milk oligosaccharide composition differs between donor milk and mother’s own milk in the NICU. J. Hum. Lact..

[66] Kiu R., Darby E.M., Alcon-Giner C., Acuna-Gonzalez A., Camargo A., Lamberte L.E., Phillips S., Sim K., Shaw A.G., Clarke P. (2025). Impact of early life antibiotic and probiotic treatment on gut microbiome and resistome of very-low-birth-weight preterm infants. Nat. Commun..

[67] Neumann C.J., Mahnert A., Kumpitsch C., Kiu R., Dalby M.J., Kujawska M., Madl T., Kurath-Koller S., Urlesberger B., Resch B. (2023). Clinical NEC prevention practices drive different microbiome profiles and functional responses in the preterm intestine. Nat. Commun..

[68] Lynch L.E., Lahowetz R., Maresso C., Terwilliger A., Pizzini J., Hebib V.M., Britton R.A., Maresso A.W., Preidis G.A. (2025). Present and future of microbiome-targeting therapeutics. J. Clin. Investig..

[69] Chen X., Mendes B.G., Alves B.S., Duan Y. (2023). Phage therapy in gut microbiome. Prog. Mol. Biol. Transl. Sci..

[70] Neu J., Pammi M. (2018). Necrotizing enterocolitis: The intestinal microbiome, metabolome and inflammatory mediators. Semin. Fetal Neonatal Med..

[71] Pammi M., Aghaeepour N., Neu J. (2023). Multiomics, artificial intelligence, and precision medicine in perinatology. Pediatr. Res..

[72] Maheshwari A., Schelonka R.L., Dimmitt R.A., Carlo W.A., Munoz-Hernandez B., Das A., McDonald S.A., Thorsen P., Skogstrand K., Hougaard D.M. (2014). Cytokines associated with necrotizing enterocolitis in extremely-low-birth-weight infants. Pediatr. Res..

[73] Hameedi S.G., Wright J.G., Schafer C.G., Sbragia L., Olutoye O.O. (2025). Serum intestinal fatty acid binding protein is elevated during feeding advancement in necrotizing enterocolitis. Pediatr. Res..

[74] Qu Y., Xu W., Han J., Zhou W., Wu H. (2020). Diagnostic value of fecal calprotectin in necrotizing enterocolitis: A meta-analysis. Early Hum. Dev..

[75] Gürünlüoğlu K., Dündar M., Ünver T., Turgut H., Gürünlüoğlu S., Akpınar N., Ateş H., Özdemir R., Yıldız T., Demircan M. (2025). Selenium deficiency is functionally linked with the molecular etiopathogenesis of necrotizing enterocolitis (NEC). Funct. Integr. Genom..

[76] Lei L., Jing M., Yingce Z., Pei Z., Yun L. (2023). Selenium deficiency causes oxidative stress and activates inflammation, apoptosis, and necroptosis in the intestine of weaned calves. Metallomics.

[77] El Manouni El Hassani S., Frerichs N.M., Berkhout D.J.C., Nijsen T., Knobel H.H., Weda H., Xu M., Long X., Wijnoltz L., van Weissenbruch M.M. (2025). Longitudinal fecal microbiota and volatile metabolomics preceding necrotizing enterocolitis in preterm infants: A case-control study. Sci. Rep..

[78] Tian B., Zhang Y., Deng C., Guo C. (2022). Efficacy of Probiotic Consortium Transplantation on Experimental Necrotizing Enterocolitis. J. Surg. Res..

[79] Wala S.J., Sajankila N., Ragan M.V., Duff A.F., Wickham J., Volpe S.G., Wang Y., Conces M., Dumbauld Z., Purayil N. (2023). Superior performance of biofilm versus planktonic Limosilactobacillus reuteri in protection of the intestines and brain in a piglet model of necrotizing enterocolitis. Sci. Rep..

[80] Huang Y., Niu W., He Y., Zhuang X., Qu Z., Lan X., Zheng C., Lin Z., Xin F., Li D. (2026). Flash nano-encapsulation of probiotics: Bolstering environmental resistance and intestinal colonization to prevent necrotizing enterocolitis. J. Control. Release.

[81] Offersen S.M., Mao X., Spiegelhauer M.R., Larsen F., Li V.R., Nielsen D.S., Aunsholt L., Thymann T., Brunse A. (2024). Fecal virus-like particles are sufficient to reduce necrotizing enterocolitis. Gut Microbes.

[82] Yi C., Chen J., She X. (2024). The emerging role of the gut virome in necrotizing enterocolitis. Heliyon.

[83] Javaudin F., Latour C., Debarbieux L., Lamy-Besnier Q. (2021). Intestinal Bacteriophage Therapy: Looking for Optimal Efficacy. Clin. Microbiol. Rev..

[84] Cassir N., Simeoni U., La Scola B. (2016). Gut microbiota and the pathogenesis of necrotizing enterocolitis in preterm neonates. Future Microbiol..

